# IL-6 Mediated Degeneration of Forebrain GABAergic Interneurons and Cognitive Impairment in Aged Mice through Activation of Neuronal NADPH Oxidase

**DOI:** 10.1371/journal.pone.0005518

**Published:** 2009-05-13

**Authors:** Laura L. Dugan, Sameh S. Ali, Grigoriy Shekhtman, Amanda J. Roberts, Jacinta Lucero, Kevin L. Quick, M. Margarita Behrens

**Affiliations:** 1 Division of Geriatric Medicine, Department of Medicine, University of California San Diego, La Jolla, California, United States of America; 2 Department of Neurosciences, University of California San Diego, La Jolla, California, United States of America; 3 Mouse Behavioral Assessment Core, The Scripps Research Institute, La Jolla, California, United States of America; Julius-Maximilians-Universität Würzburg, Germany

## Abstract

**Background:**

Multiple studies have shown that plasma levels of the pro-inflammatory cytokine interleukin-6 (IL-6) are elevated in patients with important and prevalent adverse health conditions, including atherosclerosis, diabetes, obesity, obstructive sleep apnea, hypertension, and frailty. Higher plasma levels of IL-6, in turn, increase the risk of many conditions associated with aging including age-related cognitive decline. However, the mechanisms underlying this association between IL-6 and cognitive vulnerability remain unclear.

**Methods and Findings:**

We investigated the role of IL-6 in brain aging in young (4 mo) and aged (24 mo) wild-type C57BL6 and genetically-matched IL-6^−/−^ mice, and determined that IL-6 was necessary and sufficient for increased neuronal expression of the superoxide-producing immune enzyme, NADPH-oxidase, and this was mediated by non-canonical NFκB signaling. Furthermore, superoxide production by NADPH-oxidase was directly responsible for age-related loss of parvalbumin (PV)-expressing GABAergic interneurons, neurons essential for normal information processing, encoding, and retrieval in hippocampus and cortex. Targeted deletion of IL-6 or elimination of superoxide by chronic treatment with a superoxide-dismutase mimetic prevented age-related loss of PV-interneurons and reversed age-related cognitive deficits on three standard tests of spatial learning and recall.

**Conclusions:**

Present results indicate that IL-6 mediates age-related loss of critical PV-expressing GABAergic interneurons through increased neuronal NADPH-oxidase-derived superoxide production, and that rescue of these interneurons preserves cognitive performance in aging mice, suggesting that elevated peripheral IL-6 levels may be directly and mechanistically linked to long-lasting cognitive deficits in even normal older individuals. Further, because PV-interneurons are also selectively affected by commonly used anesthetic agents and drugs, our findings imply that IL-6 levels may predict adverse CNS effects in older patients exposed to these compounds through specific derangements in inhibitory interneurons, and that therapies directed at lowering IL-6 may have cognitive benefits clinically.

## Introduction

Multiple studies have found that circulating markers of inflammation, including interleukin-6 (IL-6), are increased in older individuals[1]–[3] and are strongly associated with enhanced risk of disease and disability[3]. Longitudinal studies on centenarians and long-lived human populations indicate that longevity is inversely correlated with plasma levels of IL-6[2], [4], and conversely, that higher plasma levels of this interleukin increase the risk of many conditions associated with aging[5], including age-related cognitive decline[3], [6]–[8]. In recent epidemiologic studies, peripheral IL-6 levels showed an inverse correlation with cognitive function in aged subjects[3], [6], with chronic elevation of peripheral IL-6 associated with mild cognitive deficits[7], even in apparently healthy community-living older adults[8]. Elevated circulating IL-6 also enhances risk of stroke and dementia in older individuals[9] and has been shown to predict future cognitive decline[6]. Despite this body of literature, the specific mechanisms which link peripheral IL-6 and central nervous system impairment are still largely unknown.

In the periphery, IL-6 is well characterized as a pleiotropic cytokine with trophic effects on T-cells and other immune cells (reviewed in [2], [3], [10]). IL-6 also acts as an important pro-inflammatory cytokine and key mediator of the acute phase response, including induction of C-reactive protein (CRP). Canonical IL-6 signaling involves binding of IL-6 to its cognate receptor (IL-6R)[11] to cause dimerization of two gp130 proteins, which in turn activate intracellular signaling and transcriptional regulation through the Jak/STAT (janus kinase/signal transducers and activators of transcription) pathway, and the transcription factor SOCS3 (suppressor of cytokine signaling-3). An alternative signaling pathway involves cleavage of the full-length membrane-bound IL-6R to produce the soluble sIL-6R, which binds to IL-6 and can then activate gp130 signaling directly, even in cells lacking membrane IL-6 receptors(reviewed in [2]). Our understanding of the biology of IL-6 in the nervous system is more limited. Brain expression of IL-6 has been shown to mediate the sickness behavior, a syndrome of neurocognitive changes in response to acute infection which includes lethargy, confusion, and cognitive deficits[12]. Emerging evidence, however, suggests a more complex role for brain IL-6 in CNS function, including modulation of glutamatergic neurotransmission[13], [14].

We have recently reported in a mouse model of schizophrenia that administration of the dissociative anesthetic and drug-of-abuse, ketamine, to young mice increased brain expression of IL-6 and NADPH oxidase (Nox2)[15]. Nox2, originally described as the neutrophil respiratory burst oxidase, generates the reactive oxygen species (ROS), superoxide, through one-electron reduction of oxygen by NADPH. Recent studies on genetically modified mice suggest that elevated superoxide in brain may be a critical mediator of age-related cognitive deficits[16]. Overexpression of the cytosolic superoxide dismutase (SOD1), or the extracellular SOD (EC-SOD), but not the mitochondrial MnSOD (SOD2), reverses age-associated impairment of long-term potentiation (LTP), and prevents loss of spatial memory[16]. In addition, aged *gp91^phox−/−^* mice, which are deficient in Nox2, have better cognitive performance on memory tasks than age-matched wild-type controls[17]. It is plausible, therefore, that a cytosolic and/or extracellular source of superoxide production such as Nox2 might be an important mediator of age-related cognitive deficits.

Nox2 activation in neurons can result in dysfunction of the fast-spiking, parvalbumin (PV)-expressing subpopulation of inhibitory GABAergic interneurons[18], required for coordinated firing of excitatory neurons in cortex and hippocampus, and known to be important for the encoding and retrieval of hippocampal-dependent memory[19], [20], as well as for the generation of spatial and temporal cues. These interneurons are also involved in attentional-shift abilities, a function attributed to the pre-frontal cortex[21]. Age-associated loss of cortical and hippocampal GABAergic function has been reported for rats, primates, humans, and dogs[22]–[25], and is believed to contribute to aging-related cognitive deficits. However, the processes underlying these age-related changes in GABAergic inhibitory systems are not clear. In the present study, we wished to determine whether IL-6 is a mediator of age-related changes in brain, and if so the underlying mechanisms that are regulated by IL-6.

## Results

### Aging increases IL-6 in plasma and brain

**Figure 1:**
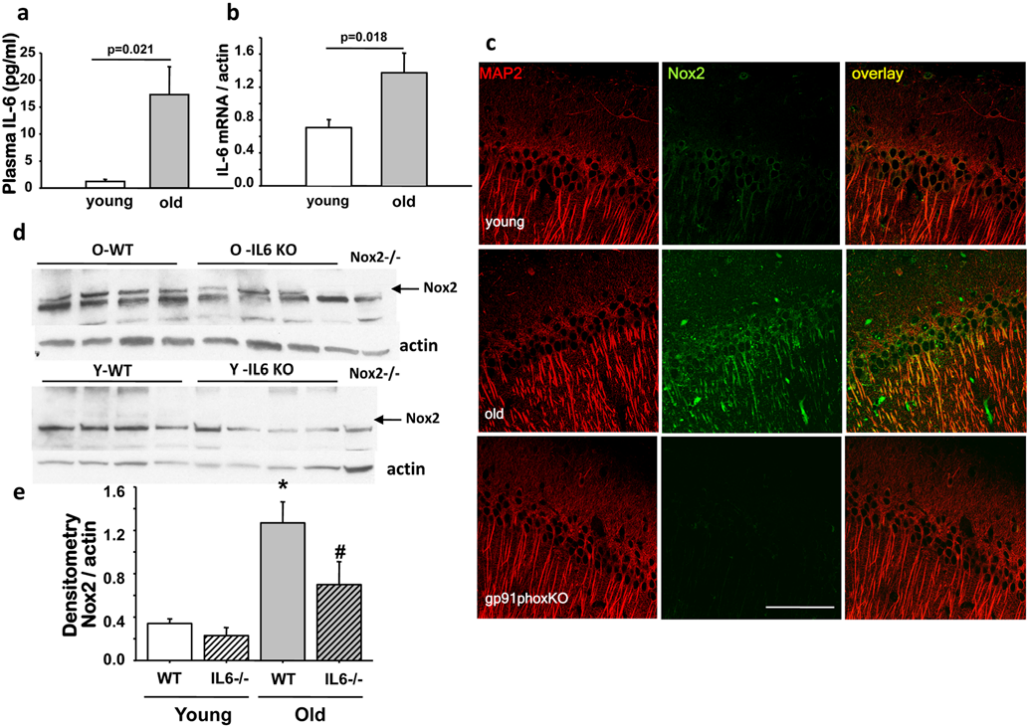
Aging increases plasma and brain levels of IL-6, and induces Nox2 protein expression which is blocked by targeted deletion of IL-6. (a) Plasma IL-6 was measured by ELISA in young (4 month) and old (24 month) C57BL6 mice and was significantly increased in old mice; n = 4, mean±SEM (F_(1,6)_ = 9.530, *P* = 0.021). (b) IL-6 mRNA expression was determined by real-time PCR in brain and was also increased significantly in old animals; n = 4, mean±SEM (F_(1,6)_ = 10.288, *P* = 0.018). (c) Confocal imaging of brain slices prepared from young (4 months) and old (23 months) mice depicting Nox2 (green) and MAP2 (red) immunoreactivity in the CA1 hippocampal region. Nox2 expression was increased in old animals with respect to young, whereas this staining was absent in slices prepared from Nox2-deficient (*gp91phox* -/-, 12 months old) mice. Bar = 100 µm. For immunofluorescence detection AlexaFluor 488 and 568 conjugated secondary antibodies were used. Merged images show co-localization in yellow. (d, e) Western blot analysis of brain protein extracts obtained from young and old wild-type and IL-6-/- mice showed increased Nox2 expression with age which was significantly lower in old IL-6-/- mouse brain (n = 4, p<0.05, mean±SEM). Expression in young animals, either WT or IL-6-/-, was below detection levels. Lack of the Nox2 band in brain proteins extracted from an old *gp91phox-/-* mouse (far right lane) confirmed specificity of the anti-Nox2 antibody and location of Nox2- specific band. N = 4 animals per age per genotype (F_(3,12)_ = 9.704, *P* = 0.002, *young versus old, *P*<0.05; ^#^ old versus old-*IL6-/-*, *P*<0.05 by Tukey's).

Multiple clinical studies have found that plasma IL-6 levels are significantly elevated in older humans[3], with nearly undetectable levels in young adults and levels generally >5 pg/ml in older subjects. To determine whether similar changes occurred in old C57BL6 mice, plasma IL-6 concentrations were measured in randomly-selected healthy old (24 mo) and young mice (4 mo) by ELISA. Levels in old mice were nearly 15-fold higher than young (Fig. 1a). To analyze whether increased plasma IL-6 levels would translate to elevation of the interleukin in brain, we measured brain IL-6 mRNA by RT-qPCR (Fig. 1b) and found increased levels in old C57BL6 mice, as previously found for comparably aged Balb/c mice[26].

### Increased Nox2 protein expression in brain of aged animals is attenuated in IL-6-/- mice

We have previously determined that Nox2 is the main source of neuronal superoxide production in a mouse model of schizophrenia[18]. Moreover, we recently demonstrated that IL-6 can directly induce and activate Nox2 protein expression in cultured neurons[15], but in that study in young mice, acute peripheral injection of IL-6 failed to induce brain Nox2 expression. In the context of chronically elevated peripheral IL-6 in aged animals, on the other hand, we wished to determine whether aging was associated with an increase in Nox2 expression in brain, by analyzing Nox2 protein expression in young (4 month old) and old (24 month old) mouse brain. Confocal images of double-immunostaining for Nox2 and the neuronal marker MAP2 in the CA1 region of the hippocampus demonstrated significantly higher protein expression in old animals (Fig. 1c). There were rare GFAP-positive astrocytes which also expressed Nox2. We also found almost no Nox2-expressing cells that also were positive for CD11b (activated microglia). Thus, more than 95% or all Nox2-expressing cells in aged mice were Map2-postive neurons. The specificity of the antibody was assessed by staining of equivalent brain regions from Nox2-deficient (*gp91^phox^-/-*) animals (Fig. 1c bottom panels). To quantify the age-related increase in Nox2 expression, and assess whether IL-6 was involved in this process, we determined levels of Nox2 protein in young and old wild-type (C57BL/6) and IL-6-deficient (*IL-6-/-*) animals. A substantial increase in Nox2 protein was observed in extracts prepared from old wild-type mice, which was absent in similar age IL-6-deficient animals (Fig. 1d, e).

### Age-related increased superoxide production in brain *in vivo* is attenuated by inhibition of NADPH oxidase or by treatment with a superoxide dismutase mimetic, and is emulated by increased levels of plasma IL-6

**Figure 2:**
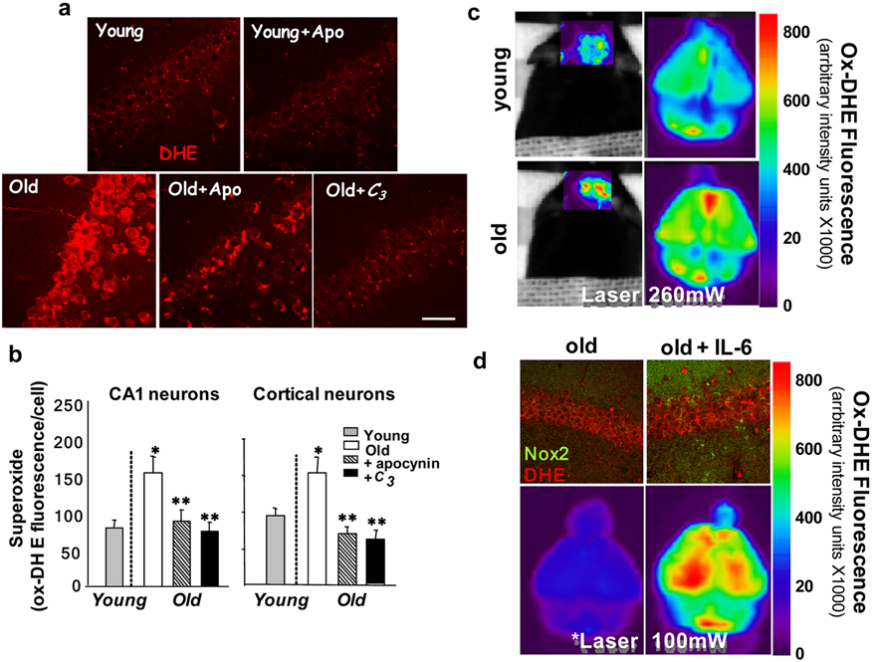
Imaging of *in vivo* superoxide production in brain of young and old animals: role of Nox2 and IL-6. (a) Confocal imaging of *in vivo* DHE oxidation reveals that superoxide-mediated DHE oxidation is increased in neurons in old mice, and this increase is blocked by the NADPH oxidase inhibitor, apocynin, or by the SOD mimetic, *C_3_*. Young (4 mo) and old (24 mo) mice were treated orally for 7 days with the Nox inhibitor, apocynin, or for 21 days with the SOD mimetic, *C_3_*. (b) Quantitative analyses of fluorescence in hippocampal CA1 and cortex were performed as described in detail in the methods section. Images were obtained using a 40× WI objective. Values are mean±SEM, n = 3. Significance was determined by ANOVA: for CA1 (*old versus young, *P* = 0.01;**old versus old+apocynin (*P* = 0.02) or old+*C_3_* (*P* = 0.01) and cortex (Ctx) (*old versus young, *P* = 0.01;**old versus old+apocynin (*P* = 0.01) or old+*C_3_* (*P* = 0.006) by Tukey's. (c) Live animal imaging of DHE oxidation using a GE eXplore Optix™-MX2 whole animal scanner also demonstrates increased DHE oxidation in brain of old (24 mo) relative to young (4 mo) mice. Fluorescence images of brain through intact skull and skin are shown on the left , and fluorescence intensity from ox-DHE is mapped onto a linear pseudocolor scale. Images to the right are *ex vivo* scans of the same brains showing greater anatomical detail, including fluorescence from the cortical hemispheres and cerebellum. (d) Repeated injections of IL-6 on two consecutive days (5 µg/kg) in old mice induced Nox2 expression (top images, green), and produced a small increase in DHE oxidation (red). However, repeated injections of IL-6 every 12 h (5 µg/kg, times three) to produce sustained systemic levels resulted in a dramatic increased in ox-DHE fluorescence compared to saline-injected old controls (bottom images). Note that the laser excitation power was decreased from 260 mW in (c) to 100 mW (d) to avoid saturating the camera in the scanner.

To confirm that elevation of brain IL-6 and induction of Nox2 described above led to increased Nox2-dependent superoxide production *in vivo,* we assessed superoxide levels using confocal imaging of dihydroethidium (DHE) oxidation to its fluorescent product (ox-DHE) in fixed brain slices prepared from DHE-injected young mice, and from DHE-injected old mice that had been treated for 7 days prior to DHE with either no drug, or with the NADPH oxidase inhibitor, apocynin, in their drinking water. Fluorescence from oxidized DHE in neurons from cortex and hippocampus was quantified as previously described[18], [27]. Increased neuronal superoxide production observed in old animals was significantly attenuated by apocynin treatment (Fig. 2a, b), strongly supporting the role of Nox2 in this increase. In addition, the requirement for IL-6 in this increase was confirmed by showing that old *IL-6-/-* mice have significantly lower superoxide levels compared to old wild-type mice (Fig. S1). We also determined that systemic administration of the SOD-mimetic, *C_3_*, for three weeks was sufficient to reduce ongoing superoxide production in aged wild-type animals (Fig. 2a and b). DHE oxidation was also evaluated in live mice using a GE eXplore Optix whole animal fluorescence scanner, which allows visualization of DHE oxidation in the intact animal. This technique also demonstrated increased superoxide production in old versus young mice, as well as in mice injected directly with IL-6 (Fig. 2c).

We next determined that increasing plasma IL-6 levels by direct peripheral injection of IL-6 enhances Nox2 expression and superoxide production in brain. Repeated intraperitoneal injection of IL-6 (5 µg/kg i.p. every 12 hours times 3) in mice significantly increased expression of Nox2 protein in brain (Fig. 2d, top) and resulted in substantially higher superoxide levels, as documented by DHE oxidation (Fig. 2d, bottom).

### Effects of IL-6 and IL-6 deficiency on Nox2 activity: oximetry and EPR spectroscopy in synaptosomes from IL-6-treated and IL-6-/- mice

**Figure 3:**
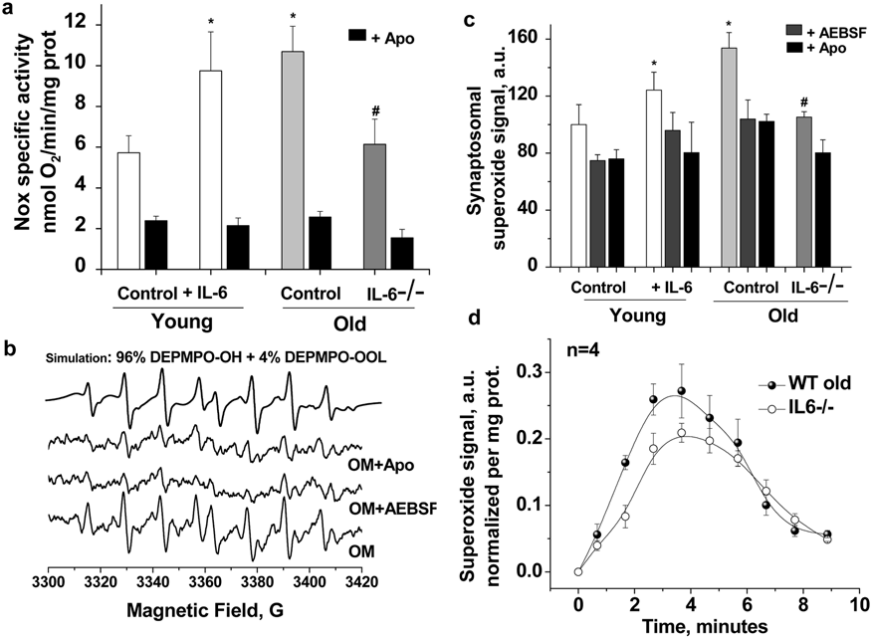
Increased synaptosomal Nox-2 activation and superoxide production in old wild-type mice is absent in old IL-6-/- mice; determination by electrochemical oxygen consumption analysis and EPR spin-trapping spectroscopy. (a) NADPH-induced oxygen consumption is significantly decreased by the Nox inhibitor apocynin (300 µM, black bars). Oxygen consumption by synaptosomal Nox from the cortex of young (3–4 M, n = 9) and old (23–24 M, n = 9) animals at 37°C was induced by the addition of 5 mM NADPH to samples containing 2–5 mg synaptosomal protein. The observed Nox specific activity was significantly higher in IL-6 injected young animals (white bars, p = 0.04, n = 5) and in old brains (light gray bar, p = 0.01, n = 9) and was significantly lower in *IL-6-/-* old mice (dark gray bar, p = 0.04, n = 4). IL-6 treatment: Two IL-6 i.p. injections (5 mg/kg), separated by 12 hrs were administered to young and old animals before the groups were sacrificed 12 hrs later for synaptosomal isolation. (b) Representative EPR spectra recorded 3 minutes after mixing 5 mM NADPH with 0.2–0.5 mg synaptosomal protein isolated from brain of old mice at 37°C with 70 mM DEPMPO in the absence or in the presence of 200 µM AEBSF or 300 µM apocynin. In each incubation, 10 mM DETC was included to inhibit the SOD enzyme, resulting in enhanced and quantifiable EPR signals. Using computer simulations we concluded that the observed signals are attributable to a mixture of adducts of DEPMPO with hydroxyl radical and lipid derived carbon centered radical and both radicals are derived from superoxide since the signals were completely abolished in the presence of SOD; not shown. (c) Quantification of the observed EPR signals accumulated over the first 3 minutes from NADPH addition, n = 4–9 per group. Superoxide yield in synaptosomes isolated from different groups follows essentially the same activity pattern seen by oxygraphy and shown in (a). The asterisks (*) designate statistical significance by ANOVA followed by Tukey test with respect to young control while (#) indicate statistical significance with respect to old control. EPR settings are described fully in methods. Data are mean ± SEM. (d) Nox-generated superoxide burst in synaptosomes isolated from *IL-6-/-* KO mice is significantly lower than that in their old control brains.

To further study the effects of IL-6 on neuronal Nox2 activation in aging, we carried out a series of studies in synaptosomes prepared from young and old wild-type and IL-6-/- mice, and in old mice injected with IL-6. We have previously reported the use of synaptosomes to carry out concurrent measurement of oxygen consumption (oximetry) and superoxide radical production (by electron paramagnetic resonance EPR)[18] allowing assessment of both Nox-dependent oxygen utilization and superoxide production in the same sample. NADPH-stimulated O_2_ consumption was significantly higher in synaptosomes prepared from old animals compared to young (F_(1,16)_ = 11.03, *P*<0.05), and this increase was completely prevented by co-incubation with the Nox inhibitor, apocynin (F_(1,16)_ = 40.95, *P*<0.0001) (Fig. 3a). Superoxide production in the same samples, assessed by EPR, was also higher in old animals (F_(1,4)_ = 24.92, *P*<0.001) (Figure 3b, c). The increase in synaptosomal superoxide production in old mice was significantly blocked by two different Nox inhibitors, aminoethyl-benzenesulfonylfluoride (AEBSF) and apocynin (Figure 3c). Statistics for 200 µM apocynin were: young: 24.1±6.5% inhibition, *P* = 0.02; old: 33.5±3.3% inhibition, *P* = 0.01, and for 200 µM AEBSF were: young: 25.2±4.1% inhibition, *P* = 0.003; old: 32.4±10.7% inhibition, *P* = 0.04. Control studies verified that neither apocynin nor AEBSF were acting as direct superoxide scavengers, and we confirmed that synaptic mitochondrial respiration and superoxide production was not altered by the conditions used to study Nox2 activity (Fig. S2).

Previous studies have reported that Nox2 and Nox4 are the only NADPH oxidase isoforms expressed in the adult mouse nervous system[28], a finding we confirmed by RT-PCR in brain of old mice as well (Dugan and Zheng, unpublished). Nox2 requires co-assembly with not only p22^phox^ , but its p47^phox^ and p67^phox^ subunits for activity, whereas Nox4 has been shown to be active in the absence of p47^phox^ and p67^phox^ [29]. Since apocynin and AEBSF block translocation and binding of p47^phox^ to the Nox-p22^phox^ complex, Nox4 is insensitive to these inhibitors, as previously reported[30]–[32]. Therefore, Nox2 represents the only apocynin-inhibitable NADPH oxidase isoform expressed in aged mouse brain. Collectively, these results suggest that synaptic Nox2 activity is significantly and specifically increased in aged mice.

To study the effect of IL-6 on synaptosomal Nox2 activity, we measured NADPH-driven O_2_ consumption and superoxide production in young mice 16 hours after repeated injection of either saline or IL-6 (5 µg/kg, i.p. on 2 consecutive days). IL-6 injected mice had significantly greater NADPH-dependent oxygen consumption (Fig. 3a, F_(1,12)_ = 5.05, p<0.01) and superoxide production (Fig. 3b, F_(1,6)_ = 15.26, p<0.01) than saline controls. We then evaluated synaptosomes prepared from young and old *IL-6-/-* mice, and found that compared to old wild-type controls, *IL-6-/-* mice had an attenuation of age-induced increases in Nox2 O_2_ consumption (Fig. 3a, (F_(1,6)_ = 15.57, p<0.01) and superoxide production (Fig. 3c, d, F_(1,6)_ = 6.34, p<0.05). In fact, Nox2 activity in old *IL-6-/-* mice was not significantly different than that observed in young wild-type mice. In Figure 3d we compare the NADPH-triggered superoxide “burst” in synaptosomes isolated from wild type old and *IL6-/-* old mice using spin-trapping EPR spectroscopy. The superoxide burst produced by synaptosomes from *IL6-/-* old brain was significantly less than from wild-type synaptosomes, confirming a key role for IL-6 on Nox-induced superoxide production at the synapse.

### Parvalbumin-positive interneurons are decreased in aged prefrontal cortex and hippocampus

**Figure 4:**
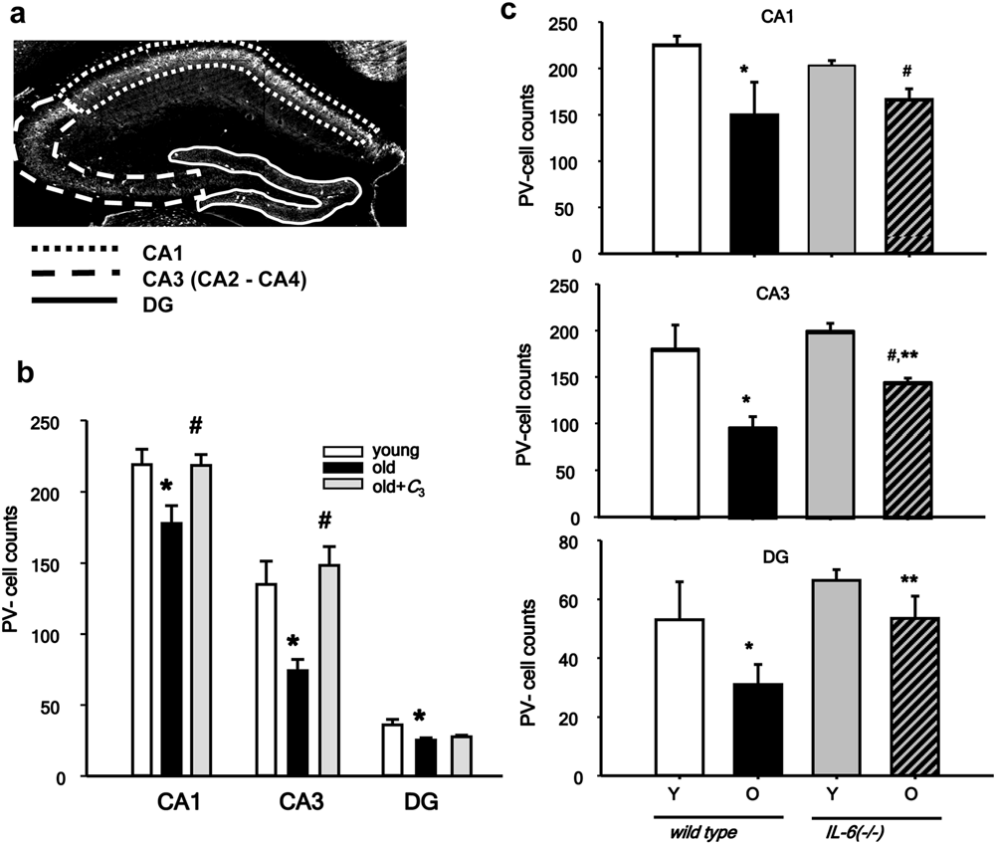
PV Interneurons are decreased with aging, but spared in IL-6 -/- mice or mice treated chronically with an SOD mimetic, *C_3_*. (a) Images, depicting the regions analyzed for PV-interneuron immunostaining in the prelimbic region (between Bregma 2.2 and 1.9), and dorsal hippocampus (between Bregmas −1.2 and −1.7). Four consecutive slices were analyzed for each animal. All cells in the regions were counted, and counts per slice were calculated as described in Methods (b) PV-cell counts in the regions of CA1, CA3 and dentate gyrus (DG) of old and old-C3 treated animals are expressed as % of their control. Aging was accompanied by a statistically significant decrease in PV-interneuron number in all regions analyzed and treatment of animals from middle age with the SOD-mimetic *C_3_* (Old-*C_3_*) prevented the reduction of PV-interneuron numbers in CA1 and CA3, but not in DG. (c) The loss of PV neurons in all of the studied regions was partially rescued in IL-6 KO mice. * indicates statistical significance with respect to young animals (P<0.05) and # indicates statistical significance with respect to old animals (P<0.05) as determined by ANOVA followed by Tukey's test. YM and OM: n = 9 animals per group; OM + *C_3_*: n = 7 animals.

**Figure 5:**
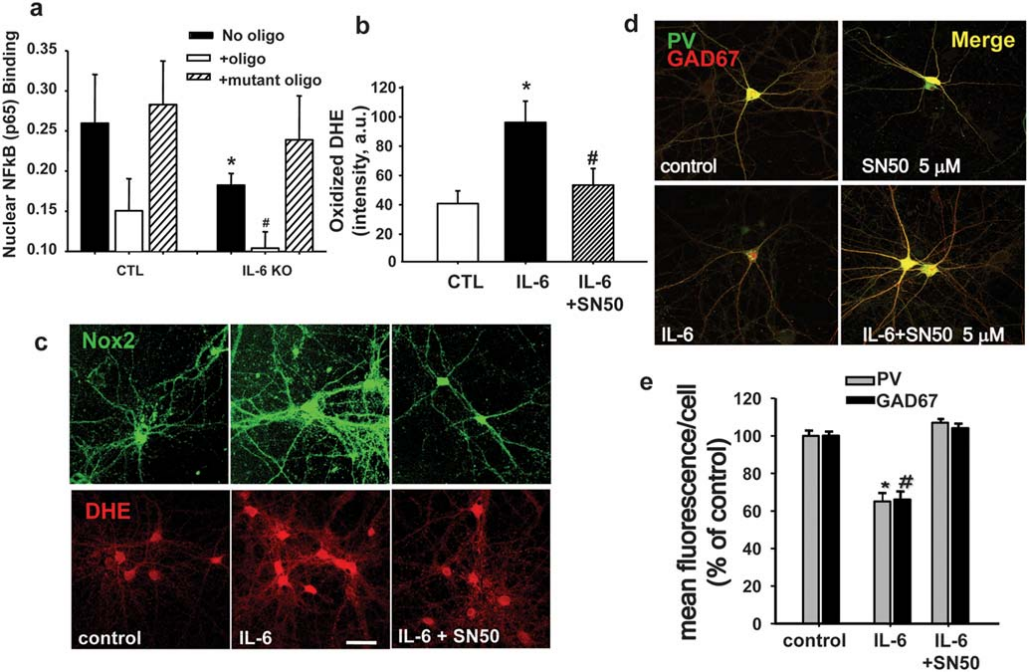
IL-6-induction of Nox2 and subsequent decrease of parvalbumin and GAD67 expression in PV-positive interneurons is NFκB-dependent. Nuclear extracts of brain samples obtained from wild-type or IL-6-deficient animals were analyzed for content of the NFkB p65 subunit by colorimetric quantification of p65 using a commercially available kit (TransAM). A high concentration of p65 was found in nuclear extracts obtained from old wild type mice (a), and this was greatly diminished in nuclear extracts obtained from similar-age IL-6 deficient mice. The specificity of the binding to wild type oligonucleotide was confirmed by competition with either sense (oligo) or mutated oligonucleotides (mutant oligo). ANOVA analysis yielded a significant effect of genotype (F_(1,18)_ = 9.414, P<0.05). (b) Application of IL-6 to neurons in culture induced Nox2 expression (b, top panel) and DHE oxidation (b, bottom panel and c) that was blocked by the NFκB inhibitor SN50. Dissociated primary neuronal cultures were treated with IL-6 (10 ng/ml) for 24 h in the absence or presence of SN50 (5 µM), an inhibitor of NFκB nuclear translocation. Dihydroethidium (DHE, 1 µg/ml) was applied for the last hour as described[18]. After fixation, cells were immunostained for Nox2. * = significant with respect to control. ^#^ = significant with respect to IL-6, F_(2,346)_ = 12.826, *P*<0.05). (d. e) SN50 also prevented the decrease in expression of parvalbumin and GAD67 induced by IL-6 exposure in PV-interneurons (*F_(2,409)_ = 234.7, *P*<0.05; ^#^F_(2,409)_ = 192.8, *P*<0.05).

Dysfunction of hippocampal mediated processes has been associated with age-related cognitive decline [33]. Spatial learning, a hippocampal form of memory, shows significant impairment in both healthy humans and rodents as they age[33], and has been correlated with disruption of glutamatergic transmission in frontal cortex and hippocampus in mice and non-human primates [34], [35]. We have recently reported that blockade of NMDA receptors by the drug-of-abuse, and dissociative anesthetic ketamine leads to increased Nox2 activity in brain, and is associated with damage to parvalbumin-positive (PV) GABAergic inhibitory interneurons[18]. The robust induction of Nox2 expression and activity we observed led us to ask whether PV inhibitory interneurons might also be selectively lost during aging, as well. We found a substantial and highly significant loss of PV-interneurons in aged hippocampus (Fig. 4), with the CA2-3 region being the most affected (20% decrease for CA1, main effect of age: F_(1,17)_ = 34.4, *P*<0.05; 45% decrease for CA2-3: main effect of age: F_(1,17)_ = 378.7, *P*<0.05; 33%, 17% decrease for DG, main effect of age: F_(1,17)_ = 20.1, *P*<0.05) (Fig. 5b), as well as a 17% decrease in prelimbic cortex (Fig. S3, *P* = 0.007 by t-test). To determine whether old mice lacking IL-6, which had lower Nox2 expression and superoxide production in brain would have preservation of PV-interneurons during aging, we carried out analysis in young and old wild-type and *IL-6-/-* mice. IL-6 deficiency attenuated the age-related loss of PV-interneurons (Fig. 4c); two-way ANOVA analysis of the genotype×age interaction showed a statistically significant effect for area CA3 (F_(1,23)_ = 23.9, *P*<0.001) and a strong trend for area CA1 (F_(1,23)_ = 3.1, *P* = 0.09). Rescue of cells in the dentate gyrus was difficult to determine because the small number of PV-interneurons in this region. To assess whether a sustained reduction in brain superoxide levels would also preserve PV-interneurons, we analyzed brain slices from mice treated chronically with an SOD mimetic, and found that *C_3_* treatment also prevented the age-related loss of PV-interneurons, as well (Fig. 4b; main effect of treatment for CA1: F_(2,22)_ = 4.9, *P* = 0.035; main effect of treatment for CA3: F_(2,22)_ = 9.3, *P* = 0.003). No tissue was available for analysis of PV-interneurons in PFC.

### Nuclear translocation of NFκB is increased in aged brain, and mediates IL-6 induction and activation of Nox2 in neurons

The activation and nuclear translocation of the transcription factor NFκB is involved in signaling mechanisms that lead to increased inflammatory cytokine expression. Conversely, IL-6 has recently been shown in an epithelial tumor cell line to activate NFκB signaling through non-canonical binding of unphosphorylated STAT3 (U-STAT3) to NFκB, releasing inhibition by IκB. The U-STAT3-NFκB dimer then translocates to the nucleus and activates a subset of NFκB-dependent genes[36]. Although both Nox2 and its p22^phox^ subunit are NFκB target genes, the question of whether IL-6 can act through NFκB to induce Nox2 or p22^phox^ has not been previously addressed. We therefore analyzed whether nuclear translocation of NFκB was elevated in forebrains of old mice, by detection of the p65 subunit using a commercially available EMSA kit (TransAM). Mouse forebrain nuclear extracts showed increased content of the p65 subunit when compared to similar extracts prepared from young animals (Fig. 5a). The age-related increase in nuclear translocation of NFκB was absent in old *IL-6-/-* mice, suggesting that NFκB is involved in the CNS effects of IL-6 (Fig. 5a). To further understand the relationship between nuclear translocation of NFκB and the activation of superoxide production by Nox2, we utilized a primary neuronal culture system we previously described as responding to ketamine with IL-6-dependent induction and activation of Nox2[15]. Exposure of these cultures to IL-6 in the absence and presence of SN50 (5 µM), a cell permeable peptide which blocks NFκB nuclear translocation[37], demonstrated that inhibiting NFκB-mediated transcriptional events completely prevented IL-6-mediated induction of Nox2 expression and superoxide production (Fig. 5b, c) and prevented the loss of GABAergic phenotype of PV-interneurons (Fig. 5d, e), as demonstrated by loss of PV and GAD67 immunoreactivity. The specificity of SN50 has previously been confirmed in these same cultures by EMSA using an inactive peptide, SN50M.[37] These results suggest that Nox2 activation/induction is dependent on NFκB-mediated transcription in neurons.

### IL-6 deficiency, or reduction of neuronal superoxide production, ameliorates impaired spatial learning in normal aging mice

**Figure 6:**
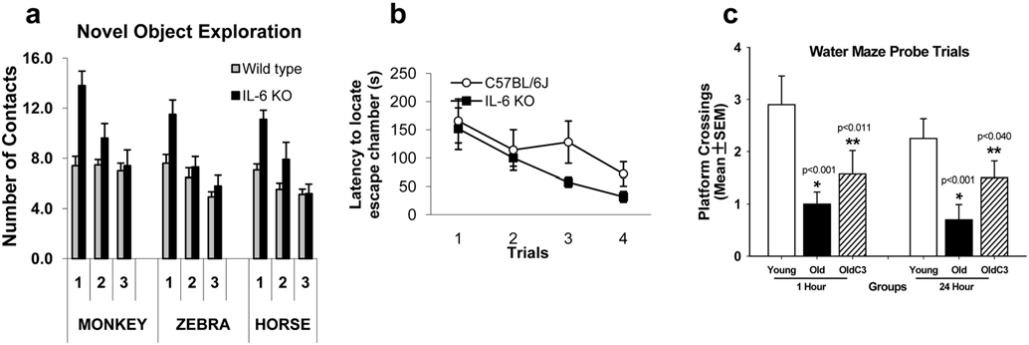
Old *IL-6-/-* mice or old mice treated with the SOD mimetic, *C_3_*, exhibit preserved spatial working memory. (a) Old *IL-6-/-* mice show improved learning performance on the Novel Object task. Old (22 month) C57BL6 and *IL-6-/-* mice (on a C57B6 background) were tested for habituation to each of three novel objects, and *IL-6-/-* mice showed significantly better performance than wild-type mice (statistics shown in Results). (b) Barne's maze escape latency (time to find the darkened chamber) showed a statistically significant decrease in latency in old *IL-6-/-* mice but wild-type mice (statistics included in results section). (c) Preservation of spatial learning and memory on the Morris water maze by chronic treatment with *C_3_*. Young (6 M), old (24 M), and old-*C_3_*-treated (24 M) male mice were tested on components of the Morris water maze as previously described[27]. Graph shows performance on the probe trial component for the specific set of male mice used in the current study. Data from the probe component of the trial are shown for the immediate (1 h) recall, and delayed (24 h) recall. *C_3_* significantly preserved performance at both time-points. Data are mean±SEM, and were analyzed by 2-Way ANOVA followed by Tukey's post-hoc test, n = 13 young, n = 10 old, n = 12 old-*C_3_*.

The PV-interneuronal GABAergic system is responsible for the control of cortical output and the generation of gamma oscillations, involved in temporal-encoding and storage/recall of information required for the correct functioning of working memory processes[38]. To analyze the possible correlation between the number of PV-interneurons in the old brain and cognitive ability, we subjected old animals to a series of simple memory tasks and compared their performance to that of similar age animals deficient in IL-6 production. Short term spatial memory recognition was tested by analyzing habituation to the spatial distribution of objects in an open field (novel object exploration task, Fig. 6a). Three objects were used for this task: a monkey, a zebra and a horse, for which the mice showed equal preference. There were significant overall genotypic differences in contacting the objects (monkey: F_(1,23)_ = 8.6, *P*<0.01; zebra: F_(1,23)_ = 5.9, *P*<0.05; and horse: F_(1,23)_ = 8.7, *P*<0.01) with IL-6-deficient animals making more overall contacts than wild-type mice. In all cases there were significant effects of trial, with decreases in contacts over trials. In addition, there were significant genotype×trial interactions in exploration of the monkey (F_(2,46)_ = 15.1, *P*<0.0001), horse (F_(2,46)_ = 5.8, *P*<0.01), and moderately for the zebra (F_(2,48)_ = 3.1, *P* = 0.05). IL-6 deficient mice showed a greater degree of habituation to object exploration than wild-type mice (Figure 6a). Indeed, overall exploration was higher in IL-6 deficient mice, but there was no floor effect limiting habituation in wild-type mice. Thus, these data suggest greater object recognition memory formation in IL-6 deficient mice. In the Barnes maze test, latencies to locate the escape chamber over the first 4 days of training were analyzed. This is the phase of the experiment where the strategy for escaping is developing and there is typically a significant decrease in escape latencies observed across this time in young mice. In this case, the aged wild-type mice did not display a significant decrease in escape latencies across the first 4 trials (F_(3,27)_ = 1.4, *P* = 0.25); whereas the IL-6 deficient mice did (F_(3,27)_ = 5.0, *P*<0.01) Fig. 6b). This also suggests greater spatial memory consolidation in *IL-6-/-* mice. To rule out possible differences in motor ability between genotypes we evaluated locomotor activity and found no baseline differences between genotypes.

Rescue of PV-interneurons in hippocampus was observed not only in IL-6 deficient mice, but in mice treated long-term treatment with an SOD mimetic (*C_3_*). Therefore, to explore the relationship between preservation of PV-interneurons and hippocampal forms of memory in *C_3_*-treated mice, we analyzed data from the probe component of the Morris water maze for the specific mice used for the PV-interneuron counts. Mice were tested on the Morris water maze as previously described[27]. The number of platform crossings over the area where the removed platform had been located was measured (higher score is better performance). Old males had significantly fewer platform crossings than young males, indicating that spatial memory is reduced in old males (Fig. 6c), On the other hand, aged *C_3_*-treated mice performed significantly better than wild-type old mice, showing that treatment with the SOD mimetic preserves spatial memory in aged mice. Data were analyzed by 2-way ANOVA and Tukey's post-hoc test.

## Discussion

Increased plasma IL-6 is an independent risk factor for many diseases associated with aging, including type 2 diabetes, atherosclerosis, hypertension, frailty, and cardiovascular ischemia, and is a significant predictor of delirium, psychosis, stroke, transition from mild cognitive impairment (MCI) to Alzheimer's disease (AD)[3], [7], and progression of AD in older individuals[2], [3]. Recent studies also suggest that even in healthy older adults without MCI or AD, circulating IL-6 is inversely correlated with cognitive performance, and that there is a linear relationship between elevated plasma IL-6 and poor cognitive function. Because of this consistent association between higher plasma IL-6 and adverse health outcomes, IL-6 was named “the gerontologist's cytokine” by William Ershler, who speculated that this cytokine regulates a key aging pathway in man[39]. However, the question of whether IL-6 is primarily a marker or is mechanistically integral to age-related nervous system dysfunction has not been established.

We found that expression of Nox2, the respiratory burst oxidase, was significantly increased in neurons throughout the hippocampus and cortex in aged mouse brain, as documented by immunocytochemistry, Western blot analysis, and mRNA (data not shown). Increased Nox2 protein was associated with enhanced Nox2 activity and superoxide production in neurons and in synaptosomes, and in fact, Nox2 appeared to be the major source of increased cytosolic and synaptosomal superoxide production in old animals. This was supported by results showing that systemic administration of the Nox inhibitor, apocynin reduced superoxide production in neurons *in vivo* to levels similar to those in young animals. Moreover, two chemically distinct Nox inhibitors, apocynin and AEBSF, fully blocked the age-related increase in Nox2 activity (O_2_ consumption) in synaptosomes prepared from old animals, and apocynin reduced synaptosomal superoxide production to levels in young synaptosomes, as well. Our data further indicated that Nox2 is tonically active in neurons *in vivo*, as evidenced by ongoing neuronal superoxide production in normal awake mice which was blocked not only by apocynin, but by oral treatment with the SOD mimetic, *C_3_*. Nox2 was also found to be constitutively active in synaptosomes; Nox2-dependent O_2_ consumption and superoxide production in synaptosomes was detected immediately after addition of the substrate, NADPH, and in contrast to a number of other studies which have required agents, such as phorbol esters, to induce assembly of the Nox2 complex, required no other additions to observe O_2_ consumption and superoxide generation. These results suggest that in neurons and at the synapse, Nox2 is in the assembled, superoxide-capable form, and is responsible for a sustained increase in neuronal and synaptic superoxide production in the aged brain. Thus, we believe this to be the first report that Nox2 is the major source of neuronal and synaptic superoxide production in normal aging brain.

There has been debate over whether apocynin inhibits the p47^phox^-dependent Nox isoforms (Nox1, Nox2, Nox4, and Nox5), with a recent paper suggesting that in vascular smooth muscle cells, apocynin may be acting as an antioxidant rather than a NADPH oxidase inhibitor, and that standard chemiluminescence-based assays for Nox activity may produce artifacts in the presence of apocynin [40]. However, there are also several recent studies supporting efficacy of apocynin as a Nox2 inhibitor[30], [41], reviewed in [42], with the use of appropriate controls. We have previously shown, using EPR and oximetry as measures of Nox2 activity, that apocynin is not acting as an antioxidant in our system ([15], [18]), that it lacks activity when Nox2 is not induced, and that it does not affect mitochondrial superoxide generation in the same sample [15], [18]. Of note, myeloperoxidase, which is required to convert apocynin to its active dimer, is expressed in neurons and astrocytes in brain[43]-[45].

The role of IL-6 in mediating age-related induction of neuronal Nox2 was then examined in old *IL-6-/-* mice, and in wild-type mice treated directly with IL-6. *IL-6-/-* mice lack the age-related increase in Nox2 expression and superoxide production observed in old wild-type mice. Nox-dependent superoxide production in synaptosomes was also significantly lower in old IL-6-/- mice compared to wild-type mice, and was not statistically different than superoxide production in young synaptosomes. Consistent with a direct effect of IL-6 on Nox2 expression, direct systemic injection of IL-6 into mice increased Nox2 protein and activity, confirming the ability of peripheral IL-6 to mediate increased Nox2 expression and superoxide production in brain. Live-animal imaging of *in vivo* ox-DHE fluorescence followed by confocal microfluorimetry of cell-specific DHE oxidation in slices prepared from live-imaged animals provided additional support for increased superoxide production in aging that was increased even further by IL-6 injection.

Factors responsible for peripheral IL-6 induction in aging humans include diet, hormone status, activity, and genetics[2], [3], [5], [46], [47], and while mechanisms which increase IL-6 in the nervous system are less well understood, direct entry of plasma IL-6 into brain has been shown[12]. Stress effects on the hypopituitary axis can also lead to local induction of IL-6 in brain and secondary release into blood, a potential additional source of IL-6 in both brain and plasma[3] (and references therein). Our ability to induce Nox2 in brain by direct peripheral IL-6 administration suggests that the former process may be operative in our experiments. Of note, these studies were carried out in male mice only, and given the fact that have previously reported higher CNS superoxide levels in aged female versus male C57BL6 mice[48], we speculate that gender-specific differences in inflammatory signaling in brain might contribute to this difference.

Induction of Nox2 by IL-6 in neurons was dependent on NFκB signaling. The decreased expression of Nox2 we observed in old *IL-6-/-* mice compared to wild-type mice was accompanied by a significant reduction in nuclear NFκB activity. Although IL-1β and TNFα are strong inducers of NFκB activation, expression of these two cytokines, measured by RT-qPCR, was not statistically different between old wild-type and old *IL-6-/-* mice (data not shown) suggesting that the specific loss of IL-6 expression, not changes in these other cytokines, was responsible for decreased nuclear NFκB activity in aged *IL-6-/-* mouse brain. More importantly, direct application of IL-6 to cultured neurons resulted in a significant increase in Nox-2 expression and superoxide production which was fully blocked by the NFκB translocation inhibitor, SN50. It has recently been shown in epithelial tumor cells that IL-6 activates NFκB signaling through binding of unphosphorylated STAT3 (U-STAT3) to NFκB, promoting STAT3-NFκB translocation to the nucleus via nuclear targeting sequences in STAT3, resulting in subsequent transcription of a subset of NFκB-dependent genes[36]. However, it has become increasingly clear that NFκB signaling is highly complex, and is dependent on the stimulus, tissue, cell type, and context. For example, NFκB activated by TNFα induces transcription of a panel of genes, including IL-6 and RANTES, whereas NFκB activation by notch fails to induce these two genes. Therefore, although both Nox2 and p22^phox^ are classic NFκB target genes which are induced by TNFα and IL-1β, this is to our knowledge, the first report that IL-6 acting through non-canonical NFκB signaling can directly induce Nox2 expression in neurons.

Recent work has suggested that the brain maintains a physiological range of superoxide production that is required for normal nervous system function. Perturbations of superoxide levels in either direction are associated with impaired LTP, altered glutamatergic neurotransmission, and poorer cognitive performance. Elevated levels of superoxide associated with aging have specifically been shown to mediate deficits in learning and memory[27], [49], [50]. Thus, superoxide in brain has been the focus of several recent studies on cognitive deficits in aging.

Increased neuronal superoxide production at the synapse, specifically the glutamatergic synapse, can affect a number of redox-regulated proteins important to synaptic activity and plasticity [18], [51]. Dysregulation of the activity of these proteins would in turn affect normal glutamatergic transmission, and thus cognition. We recently reported that acute (days) elevation of superoxide production in brain by ketamine caused the loss of phenotype of PV-interneurons, as demonstrated by reduced PV and GAD67 immunoreactivity, which recovered over a few days. In contrast, here we show that sustained superoxide production by Nox2 during aging produces a significant decrease in these same neurons throughout hippocampus and prefrontal cortex. There was loss of 45%, 20%, 33%, and 17% of these neurons in hippocampal CA3, CA1, DG, and prefrontal cortex, respectively, in 24 month-old mice. This decline was completely rescued by treatment of mice with the small molecule SOD mimetic, *C_3_*, from middle age. Systemic treatment with *C_3_* has been previously shown to reduce brain superoxide levels significantly. These results strongly suggest that sustained Nox-dependent superoxide production during aging may be responsible for the decline in PV-interneuron numbers. In addition, as predicted by our results showing that injected IL-6 induces brain Nox2, and that *IL-6-/-* mice lack induction of Nox2, there was preservation of these interneurons in aged *IL-6-/-* mice which was highly significant for CA3, and approached significance for CA1. While our data suggest that neurons are the primary source of Nox2-derived synaptic superoxide, and that in this model of non-pathological aging, very few astrocytes have detectable Nox2 protein or superoxide production, there is no way to rule out local Nox-dependent superoxide release by astrocytes at the synapse as contributing to the observed effects.

PV-interneurons subserve an important role in cognitive processes, and are critically positioned to modulate hippocampal and PFC-dependent cognitive processes [38]. Although PV-neuron loss was observed in all regions of hippocampus, as well as prefrontal cortex, the most pronounced decrease was in CA2-3. Both *IL-6-/-* mice and *C_3_*-treated mice showed preservation of PV-interneurons in CA3, and *C_3_* rescued interneurons in CA1, as well. The CA3a,b region of hippocampus has been shown to be critical for detection of novelty[52], spatial learning[53], and for encoding of new spatial information into short-term memory[52], [54]–[56]. Importantly, impaired spatial learning and memory is consistently reported across studies in older adults, and this is found even in the absence of a dementing illness[53], [55]. While CA1 is generally believed to be necessary for encoding of temporal but not spatial information, it is believed to be involved retrieval of spatial information previously encoded by CA3[56], [57]. Thus, the behavioral correlates of deficits in CA3 neural circuitry would be decreased novelty recognition and impaired spatial learning[58], and for CA1, decreased spatial working memory[56], [57]; and both areas are believed necessary for effective performance on the probe component of the Morris water maze. To determine whether rescue of PV-interneurons was associated with improved cognitive performance, we carried out behavioral testing, and found that aged IL-6-/- mice, which had significant preservation of PV-neurons in CA3, had significantly greater recognition of three novel objects, and then showed greater habituation to compared to wild-type controls. Habituation to a novel environment tests simple learning[52], [58] without the need for aversive stimuli, and has been shown to be impaired in animals with lesions to CA3. In addition, while aged wild-type mice showed impaired abilities in finding the escape chamber in the Barnes maze, *IL-6*-/- mice progressively found the escape chamber more quickly across trials. Thus, IL-6 deficiency appears to decrease learning deficits attributable to CA3 in aged mice. We further evaluated spatial learning and memory in aged mice treated long-term with a SOD mimetic. Mice treated chronically with *C_3_* had significant rescue of PV-interneurons in both CA1 and CA3. Consistent with the importance of these regions to spatial memory, treated mice had significantly better performance on the probe component of the Morris water maze. Of note, a virtual Morris water maze for humans has been developed[59], [60] and has revealed that older humans show a nearly identical decline in performance on the probe trial component[59], [60]. Taken together, our data suggest that loss of PV-interneurons in the hippocampus could underlie age-related cognitive deficits in older humans. Furthermore, the data suggests that decreasing IL-6 induction of Nox2, or eliminating downstream superoxide production directly can preserve cognitive performance.

Increased circulating IL-6 is a highly prevalent finding in older adults, with estimates based on the clinical literature that more than 80% of individuals over the age of 65 will have circulating levels of IL-6 greater than 5 pg/ml. This indicates that a sizeable and growing number of older individuals might be at risk for IL-6-mediated cognitive effects. In addition, our results showing that sustained elevation of peripheral IL-6 results in loss of PV-interneurons with aging provide several clinically-testable hypotheses. The PV-interneurons targeted by IL-6 represent the same interneuron population that is selectively vulnerable to injury from commonly-used general anesthetics, and this vulnerability is observed even at low doses of these agents. Thus, the use of general anesthetics in patients with chronic elevation of circulating IL-6 might be predicted to increase the risk of adverse cognitive outcomes after anesthesia. Because treatments which lower plasma IL-6 levels and/or block IL-6 signaling are currently in clinical use for other indications, these agents could be used to test idea that lowering IL-6 may preserve in the integrity of the PV-inhibitory system and prevent associated cognitive deficits. Clinical testing of the mechanisms presented here could further contribute to our broader understanding of the role of inflammatory signaling in both physiology and pathology the brain.

## Methods

### Materials

Antibodies used were as follows: anti-parvalbumin rabbit polyclonal was from Swant (Bellizona, Switzerland), and anti-GAD67 monoclonal from Chemicon (Millipore). Polyclonal anti-Nox2 was from Santa Cruz Biotechnology Inc. (Santa Cruz, California), and monoclonal anti Nox2 was from BD Transduction, or a generous gift of Mark Quinn (Montana State University). Unless otherwise stated, all reagents were from Sigma and tissue culture media from Invitrogen. The spin traps 5-(diisopropoxyphosphoryl)-5-methyl-1-pyrroline-N-oxide (DIPPMPO) and 5-(diethylphosphoryl)-5-methyl-1-pyrroline-N-oxide (DEPMPO) were from Alexis Biochemicals (San Diego, CA). The NFκB inhibitor, SN50, was from Calbiochem (San Diego, CA).

#### Maintenance of mice, and administration of dihydroethidium (DHE), apocynin, IL-6, and the SOD mimetic, C_3_

Male C57BL/6, *gp91phox(-/-)* and *IL-6(-/-)* mice were obtained from Jackson Labsat 8 weeks of age and housed in our facility until 12–15 weeks (young), or 22–26 months of age (old), when they were used for experiments. DHE was injected as described[27]. Briefly, two serial i.p. injections of freshly prepared dihydroethidium (27 mg/kg) are given at 30 minute intervals. Eighteen hours later, mice are anesthetized with inhaled halothane, and perfused intracardially with cold saline followed by 4% paraformaldehyde in PBS for immunohisto-chemistry experiments, or their brains dissected and forebrains processed for synaptosomal preparations (as described below).

Apocynin (5 mg/kg/day) was administered in the drinking water for 7 days. IL-6 injections were 5 µg/kg i.p. on 2 consecutive days as described[15], except for *in vivo* imaging, in which an additional 5 µg/kg injection was given between the first and last injection. *C_3_* was given in the drinking water at 1 mg/kg/day starting at 12 months of age as described[27] for cell counts, or for 21 days for DHE studies. All animal studies were approved by the Animal Care Program at the University of California, San Diego, and are in accordance the PHS Guide for the Care and Use of Laboratory Animals, USDA Regulations, and the AVMA Panel on Euthanasia.

#### Synaptosomal preparations

Synaptosomes were prepared from adult mouse forebrain as described[18].

Cortical neuronal cultures were prepared form cortices of Swiss Webster E15 mouse embryos and grown on poly-lysine coated coverslips with paraffin “feet” for 21 days as previously described[18].

#### RT-PCR

RNA was extracted from mouse forebrain using Trizol. After reverse transcription of mRNA (1 µg), the cDNA produced was analyzed for IL-6, IL-1β, and TNFα expression using actin as an internal control. PCR was carried out by CFAR Genomics Core at UCSD. PCR conditions were the following 45 seconds at 95°C, 15 seconds at 45°C, and 1 minute at 72°C for 35 cycles. IL-6 primers: forward: 5′-atggatgctaccaaactggat-3′ and reverse: 5′-tgaaggactctggctttgtct-3′. IL-1β primers: 5′-caaccaacaagtgatattctccatg-3′ and reverse: 5′-gatccacactctccagctgca-3′. TNFα primers: 5′-catcttctcaaaattcgagtgacaa-3′ and reverse: 5′-tgggagtagacaaggtacaaccc-3′. GAPDH primers: forward: 5′-gaacatcatccctgcctctactgg-3′ and reverse: 5′-tccaccaccctgttgctgta-3′.

### Superoxide detection in synaptosomes by electron paramagnetic resonance (EPR) spectroscopy

Detection of NADPH-dependent superoxide production in synaptosomal preparations was performed using spin-trapping EPR spectroscopy as described[15], [18]. In brief, synaptosomes (∼0.1–0.2 mg synaptosomal protein) were mixed with 70 mM DEPMPO (5-(diethylphosphoryl)-5-methyl-1-pyrroline-N-oxide) and appropriate combinations of substrates/inhibitors, and loaded into a 50 µl-glass capillary, which is inserted into the EPR cavity of a MiniScope MS200 Benchtop spectrometer, maintained at 37°C. Previous control experiments have confirmed that the EPR signals are substrate specific, acquired only in the presence of synaptosomes, and not due to redox cycling of reagents used[18]. EPR signals accumulated during the first 6 minutes after addition of NADPH were measured. To be able to detect all superoxide generated, the SOD inhibitor diethyldithiocarbamic acid (DETC; 5 mM) was included in the assay mixture. When analyzing the effects of direct addition of IL-6 on superoxide production in synaptosomes, IL-6 (100 ng/ml, final) was added to synaptosomes 5 minutes before Nox oxidase activity was initiated by addition of substrate (NADPH,5 mM final). EPR conditions were: microwave power, 5 mW; modulation amplitude, 2 G; modulation frequency, 100 kHz; sweep width, 150 G centered at 3349.0 G; scan rate, 7.5 G s^−1^, with each spectrum representing the average of 5 scans.

#### Immunocytochemistry

Fixation of neurons in culture was performed as described[61]. For dual-label immunostaining, coverslips were incubated in 2% normal goat serum (NGS) containing the following primary antibodies (Ab): anti-GAD67 monoclonal (1∶1000), anti-Nox2 rabbit polyclonal (1∶200), anti-parvalbumin rabbit polyclonal (1∶4000), and incubated for 2 h at 37°C. Specific binding was detected by incubation for 45 min at room temperature with a 1∶1000 dilution of AlexaFluor secondary Abs (568: red; 488: green).

#### Immunohistochemistry

Brains were sliced using a vibratome into 50 µm coronal sections. As previously described[27], 6 sequential slices, encompassing either the prelimbic region (from Bregma 2.0 to 1.3), or dorsal hippocampal formation, were processed by floating-section fluorescence immunohistochemistry to detect Nox2 and MAP2[18]. For PV-interneuron counting, slices were processed as before and incubated with anti-Parvalbumin antibody (1∶5000) for 48 h, washed in PBS and developed using a Vectastain ABC kit according to manufacturer's instructions

#### Confocal microscopy, live animal fluorescence imaging, and image analysis

Mounted slices or coverslips were evaluated for fluorescence under settings for 488 and 568 excitation on a LSM510 Meta multiphoton laser confocal microscope using 10× or 40× water immersion objective. Oxidized DHE fluorescence was analyzed using Ex λ 543 nm, Em λ>590 nm. Imaging for PV-interneuron counting was performed using a transmitted light. All PV-positive interneurons were counted in the regions described in Figs 5 and suppl. 2.

Image analysis of the neuronal population in primary cultures was essentially as described [61]. Briefly, coverslips are scanned to obtain 200–400 neurons (approx. 26–30 images captured per coverslip per condition using a 40× water immersion objective). Each image analyzed consists of a stack of 16 0.2 µm Z-stage images taken from the base of the neurons and across 3.2 µm depth. When analyzing PV-interneurons in particular, the coverslips are scanned to obtain images as before but all PV-interneurons on the coverslip are imaged. Experiments were performed in duplicate coverslips of neurons prepared from the same dissection across at least three different dissections.

Confocal microscope settings were maintained constant for each series of experiments so that the resulting images could be analyzed by densitometry and the treatment-dependent changes in fluorescence compared and expressed as % of control (for primary cultures) or saline (for *in vivo*) conditions. For each experiment, both in vitro and in vivo, controls (saline) were processed in parallel such that variability due to antibody batch or laser power could be avoided. Neurons in the images were then analyzed for their somatic median green and red fluorescence intensity per cell using MetaMorph (linear scale of 0–255 pixel intensity units).

Live animal imaging of oxidized DHE (ox-DHE) fluorescence in brain was carried out as described[62]. Briefly, anesthesia was induced in mice injected 16 hours previously with DHE using inhaled isofluorane. Mice were then placed in a eXplore Optix™-MX2 system whole animal scanner (Advanced Research Technologies, Inc., Montreal, Canada), and maintained on isofluorane anesthesia by nose-cone. Fluorescence images were obtained by raster-scanning a region over brain using a step size of 0.5 mm with 1 second integration time.

#### Behavioral testing

The novel object habituation test was adapted from the Novel Object/Location test as described[63]. To habituate each mouse to the testing environment, they were individually placed in an empty testing chamber (a 51 cm×51 cm×39 cm opaque Plexiglas box) for 5 minutes prior to the beginning of testing. Each mouse was then moved to a holding cage, and three novel objects (plastic toy animals glued to a 4×4 in Plexiglas base) were placed in three corners of the testing chamber. After a brief interval, the mouse was placed back into the chamber for another 5 minutes; exploration was assessed by analyzing the number of contacts, defined as nose-first approaches to within 2–4 cm of an object, with a given object in a trial, and habituation was assessed by comparing the number of contacts made with each object over the course of two subsequent trials, three in all. Between animals, the testing chamber, as well as the objects, were cleaned with AirX44 disinfectant and odor counteractant (AirX Laboratories; Folcroft, PA), to remove olfactory cues. One week following novel object exploration, the Barnes maze test was performed as described[64]. The Barne's maze was carried out as previously described[65]. The Morris water maze was carried out as described in detail[27].

#### Statistical analysis

For analysis of fluorescence, all intensity values were normalized by the mean obtained for the control treatments (medium exchange alone for cultures, saline injection for animals), processed in parallel with the experimental group, and expressed as a percent of this mean. To obtain the mean fluorescence/cell/animal, percent values were averaged across six slices from the same animal, or from the same culture plating in the case of primary cultures on coverslips. The mean fluorescence intensity/cell/animal (or per experiment in the case of primary cultures) was then used to calculate the mean and standard deviation per group. These were then used for statistical analysis using SigmaStat software. Values obtained per experiment were analyzed by one- or two-way ANOVA as indicated in each figure, followed by Tukey's post-hoc test for multiple comparisons, with significance set at *P*<0.05.

Two-way ANOVA followed by Tukey's post-hoc analysis was used to determine significance between genotypes across time for the Novel Object task and the Barnes Maze, or between ages and treatment groups for the Morris water maze, and F and *P* values for these comparisons are included in the appropriate figure legend or results section.

## Supporting Information

Figure S1 Superoxide imaging in IL-6-/- brain. Superoxide levels are substantially lower in the hippocampal CA1 and cortex regions of IL-6-/- mouse. Confocal images were acquired as described for Figure 2a.
(10.58 MB TIF)

Figure S2 Synaptosomal mitochondria. Synaptosomal mitochondrial respiration is not affected by age or IL-6 treatment. State 3 respiration was initiated by malate plus pyruvate (10 mM each) (ADP already present), state 4 respiration was measured after addition of the F0F1-ATPase inhibitor, oligomycin, and maximal respiration was measured after adding the uncoupling agent, CCCP. IL-6 treatment: Two IL-6 i.p. injections (5 mg/kg), separated by 12 hrs were administered to young and old animals before the groups were sacrificed 12 hrs later for synaptosomal isolation.
(10.03 MB TIF)

Figure S3 PV-interneurons in PFC. A statistically significant decrease in PV-positive cell counts was observed in the prelimbic region of prefrontal cortex (PFC). The region analyzed is shown at left. Values are mean±SEM, P = 0.007 by t-test.
(10.03 MB TIF)
