# Supplementary figures and images for: IL-6 Mediated Degeneration of Forebrain GABAergic Interneurons and Cognitive Impairment in Aged Mice through Activation of Neuronal NADPH Oxidase
Source: PLoS One. 2009 May 13;4(5):e5518. doi: 10.1371/journal.pone.0005518 (PMC2678193; doi:10.1371/journal.pone.0005518)

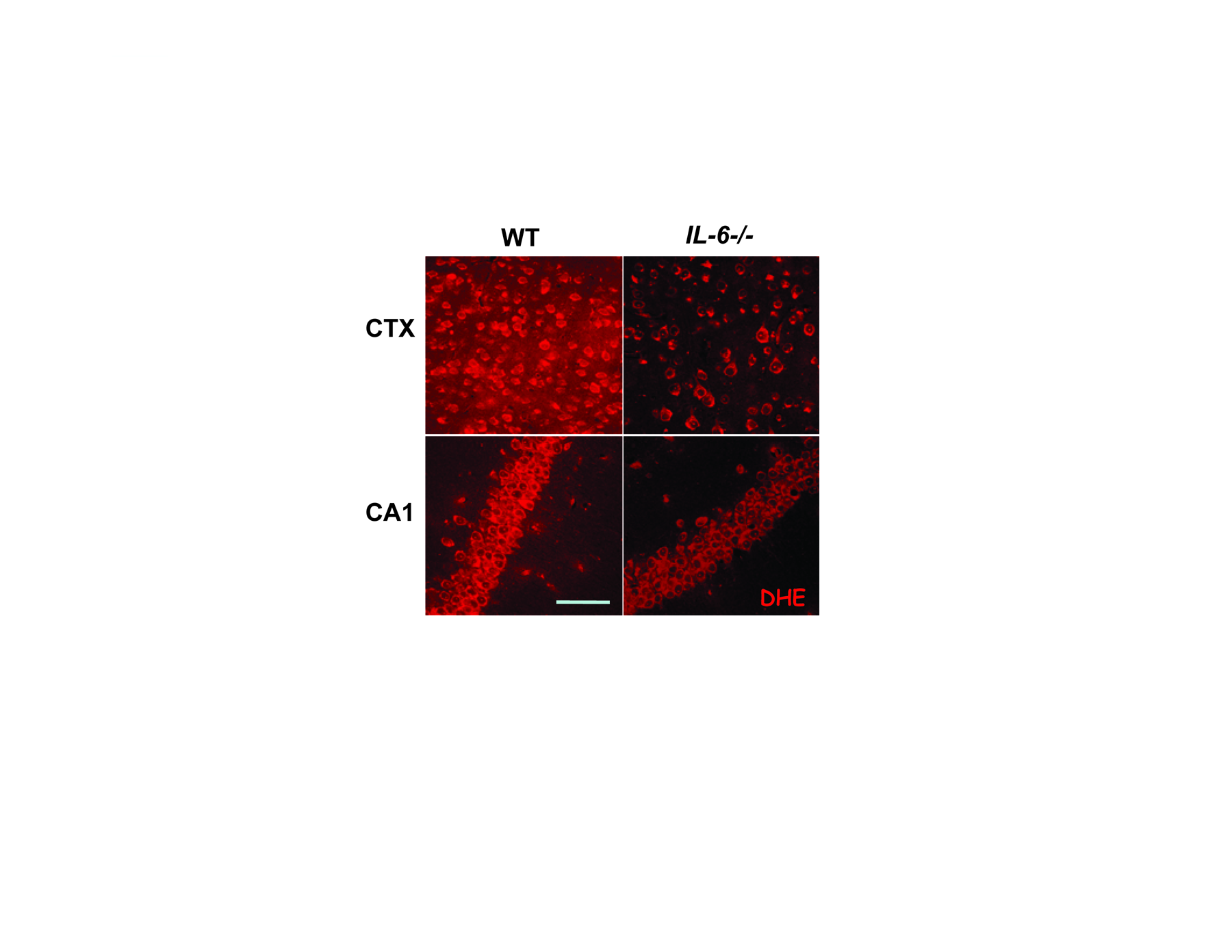

Supplement: Figure S1 — Superoxide imaging in IL-6-/- brain. Superoxide levels are substantially lower in the hippocampal CA1 and cortex regions of IL-6-/- mouse. Confocal images were acquired as described for Figure 2a. (10.58 MB TIF) [file pone.0005518.s001.tif]

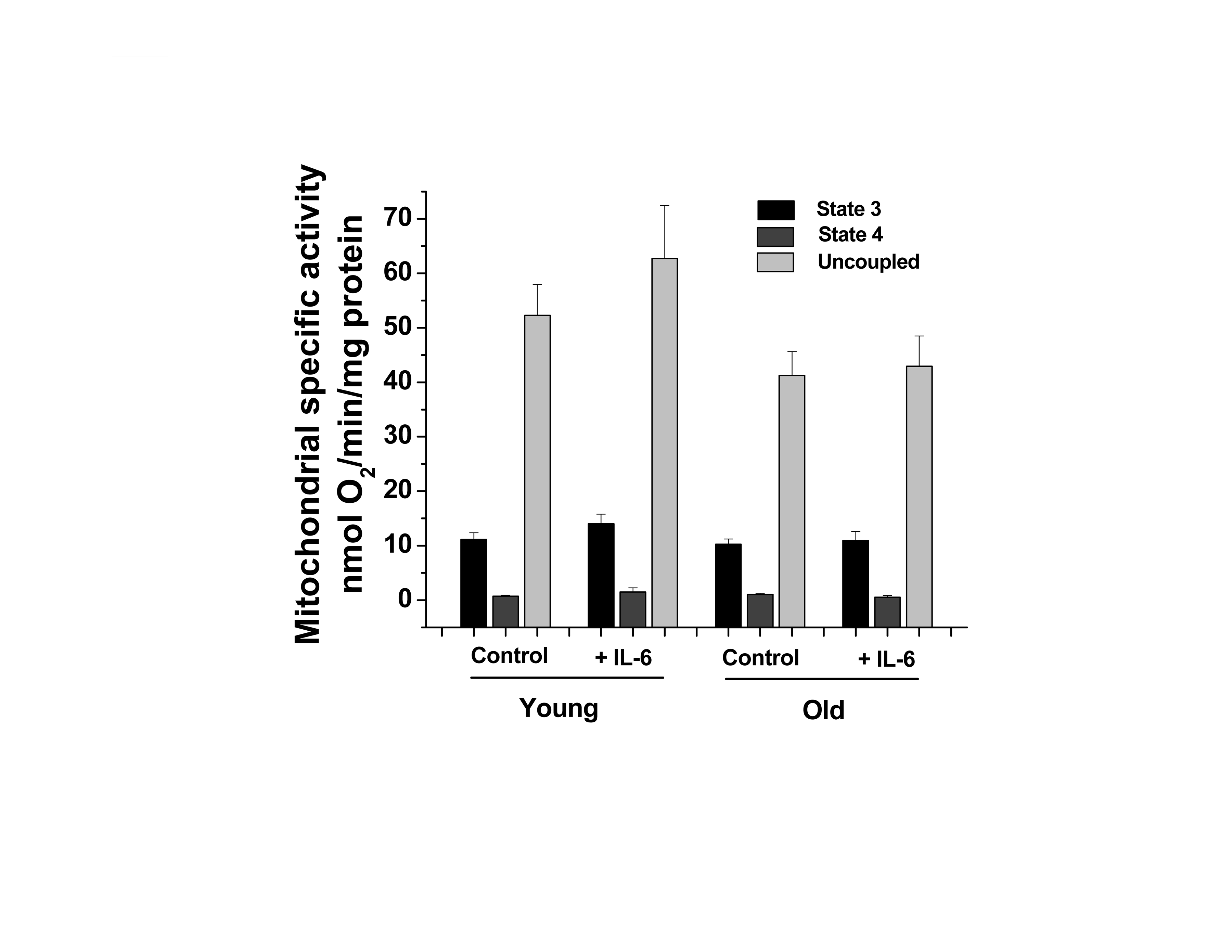

Supplement: Figure S2 — Synaptosomal mitochondria. Synaptosomal mitochondrial respiration is not affected by age or IL-6 treatment. State 3 respiration was initiated by malate plus pyruvate (10 mM each) (ADP already present), state 4 respiration was measured after addition of the F0F1-ATPase inhibitor, oligomycin, and maximal respiration was measured after adding the uncoupling agent, CCCP. IL-6 treatment: Two IL-6 i.p. injections (5 mg/kg), separated by 12 hrs were administered to young and old animals before the groups were sacrificed 12 hrs later for synaptosomal isolation. (10.03 MB TIF) [file pone.0005518.s002.tif]

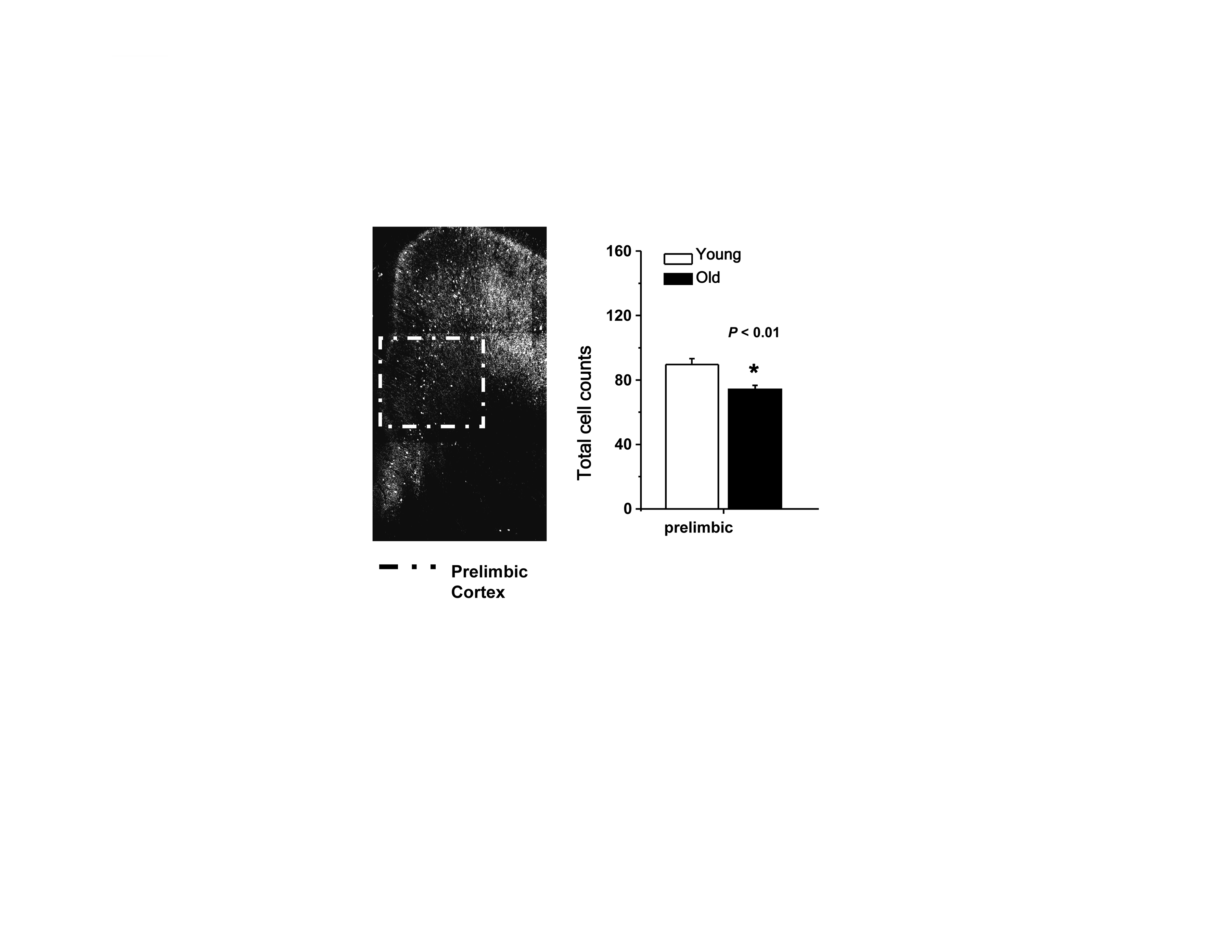

Supplement: Figure S3 — PV-interneurons in PFC. A statistically significant decrease in PV-positive cell counts was observed in the prelimbic region of prefrontal cortex (PFC). The region analyzed is shown at left. Values are mean±SEM, P = 0.007 by t-test. (10.03 MB TIF) [file pone.0005518.s003.tif]
